# dnadna: a deep learning framework for population genetics inference

**DOI:** 10.1093/bioinformatics/btac765

**Published:** 2022-11-29

**Authors:** Théophile Sanchez, Erik Madison Bray, Pierre Jobic, Jérémy Guez, Anne-Catherine Letournel, Guillaume Charpiat, Jean Cury, Flora Jay

**Affiliations:** Université Paris-Saclay, CNRS UMR 9015, INRIA, Laboratoire Interdisciplinaire des Sciences du Numérique, 91400 Orsay, France; Université Paris-Saclay, CNRS UMR 9015, INRIA, Laboratoire Interdisciplinaire des Sciences du Numérique, 91400 Orsay, France; Université Paris-Saclay, CNRS UMR 9015, INRIA, Laboratoire Interdisciplinaire des Sciences du Numérique, 91400 Orsay, France; ENS Paris-Saclay, 91190 Gif-sur-Yvette, France; Université Paris-Saclay, CNRS UMR 9015, INRIA, Laboratoire Interdisciplinaire des Sciences du Numérique, 91400 Orsay, France; UMR7206 Eco-Anthropologie, Muséum National d’Histoire Naturelle, CNRS, Université de Paris, 75016 Paris, France; Université Paris-Saclay, CNRS UMR 9015, INRIA, Laboratoire Interdisciplinaire des Sciences du Numérique, 91400 Orsay, France; Université Paris-Saclay, CNRS UMR 9015, INRIA, Laboratoire Interdisciplinaire des Sciences du Numérique, 91400 Orsay, France; Université Paris-Saclay, CNRS UMR 9015, INRIA, Laboratoire Interdisciplinaire des Sciences du Numérique, 91400 Orsay, France; SEED, U1284, INSERM, Université de Paris, 75004 Paris, France; Université Paris-Saclay, CNRS UMR 9015, INRIA, Laboratoire Interdisciplinaire des Sciences du Numérique, 91400 Orsay, France

## Abstract

**Motivation:**

We present dnadna, a flexible python-based software for deep learning inference in population genetics. It is task-agnostic and aims at facilitating the development, reproducibility, dissemination and re-usability of neural networks designed for population genetic data.

**Results:**

dnadna defines multiple user-friendly workflows. First, users can implement new architectures and tasks, while benefiting from dnadna utility functions, training procedure and test environment, which saves time and decreases the likelihood of bugs. Second, the implemented networks can be re-optimized based on user-specified training sets and/or tasks. Newly implemented architectures and pre-trained networks are easily shareable with the community for further benchmarking or other applications. Finally, users can apply pre-trained networks in order to predict evolutionary history from alternative real or simulated genetic datasets, without requiring extensive knowledge in deep learning or coding in general. dnadna comes with a peer-reviewed, exchangeable neural network, allowing demographic inference from SNP data, that can be used directly or retrained to solve other tasks. Toy networks are also available to ease the exploration of the software, and we expect that the range of available architectures will keep expanding thanks to community contributions.

**Availability and implementation:**

dnadna is a Python (≥3.7) package, its repository is available at gitlab.com/mlgenetics/dnadna and its associated documentation at mlgenetics.gitlab.io/dnadna/.

## 1 Introduction

In recent years, deep learning has been applied to biology with the hope of facilitating complex data analyses and information discovery, and methods are now flourishing in population genetics (Borowiec *et al.*, 2022). As reviewed by Sanchez *et al.* (2021), we distinguish two families: those processing many summary statistics, with fully connected or convolutional networks and those based on ’raw’ genetic data leveraging deep architectures to automatically construct informative features (e.g. Adrion *et al.*, 2020b; Battey *et al.*, 2020; 2021; Burger *et al.*, 2022; Chan *et al.*, 2018; Deelder *et al.*, 2021; Flagel *et al.*, 2019; Fonseca *et al.*, 2021; Gower *et al.*, 2021; Isildak *et al.*, 2021; Meisner and Albrechtsen, 2022; Montserrat *et al.*, 2019; Perez *et al.*, 2022; Qin *et al.*, 2022; Sanchez *et al.*, 2020; Torada *et al.*, 2019; Wang *et al.*, 2021; Yelmen *et al.*, 2021). Previous studies have made their implementations available at least for reproducibility and sometimes with a specific effort for re-usability. Even so, each of them focuses on a specific network for a specific task. Adapting them requires a careful understanding of the code and its direct modification since many options are hard-coded. This is not only error-prone but also leads to a rapid code divergence between parallel projects, accompanied by complex maintenance. The community demands flexible and rigorous tools as demonstrated by stdpopsim, a library for population genetic simulations which allows contributions from many researchers (Adrion *et al.*, 2020a). In genomics, a suite of tools has been developed to facilitate deep learning applications (e.g. Kopp *et al.*, 2020; Routhier *et al.*, 2021; Zhang *et al.*, 2021). However, none is able to handle population genetic datasets and tasks. For these reasons, we developed dnadna, Deep Neural Architectures for DNA, a task-agnostic software (in the context of population genetics) that aims at facilitating applications, development, distribution and exchanges around neural networks in the field.

## 2 Software

dnadna is a python-based software for population genetics inference that enables researchers to (i) develop new networks or re-use existing architectures, (ii) train them for a given task (regression, classification or a mix of those) and (iii) share them in such a way that users can easily apply these trained networks to their own dataset. In particular, it already implements several neural networks that have been tested for inferring demographic and adaptive histories from genetic data. Pre-trained networks can be used directly on real and simulated genetic data for the prediction step. Networks can also be re-trained on new simulations (e.g. corresponding to another species or evolutionary model) and/or to solve other tasks (e.g. classifying introgressed versus non-introgressed segments or inferring recombination). Finally, any user can implement new architectures and tasks, while benefiting from training procedure, test environment, routines that may be otherwise overlooked (such as proper preprocessing or efficient data loading), and the possibility to easily share a network to facilitate re-use and benchmarking.

**Fig. 1.**
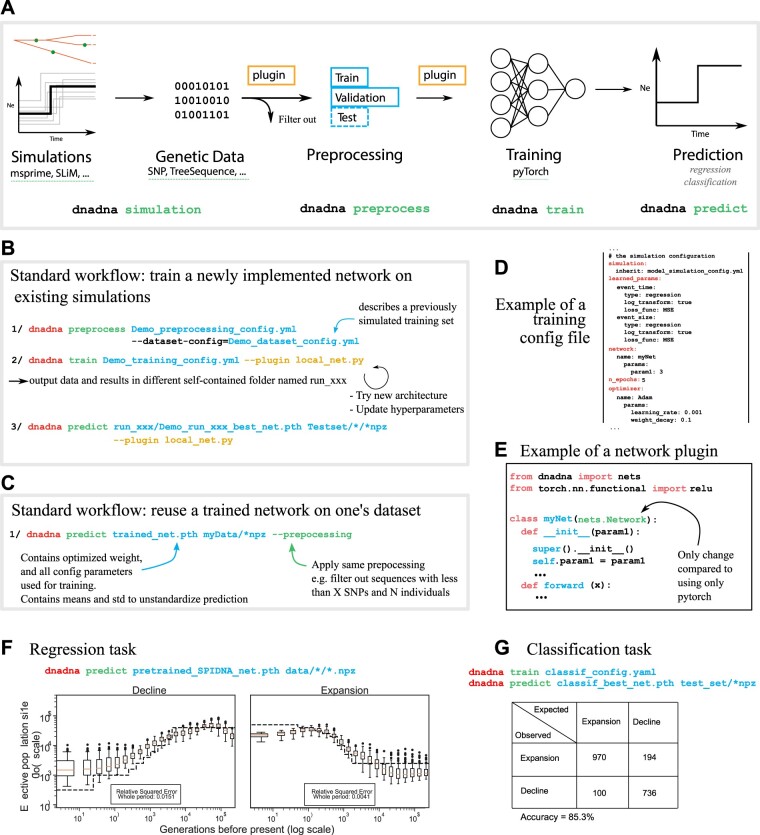
(**A**) dnadna workflow and its corresponding commands. Each step could be done as a standalone: (1) simulation of a large genetic dataset according to evolutionary scenarios and priors; genetic data type is not enforced and can be Boolean (classical single-nucleotide polymorphism (SNP) data), integer (e.g. genotype data 0/1/2) or float [e.g. local density of SNPs as in Gower *et al.* (2021) or summary statistics along the genome as in Xue *et al.* (2021)]; (2) preprocessing, mainly to filter out examples that do not fit minimal requirements and split the rest into train/validation/test sets; (3) training neural networks; (4) predicting on test or real datasets using optimized neural networks. Note that simulations can be skipped if the user already possesses a labeled dataset. Similarly, training can be skipped if the user reuses a pre-trained network. Here is a subset of options at each step: (1a) generating simulations: name of predefined scenario to be simulated and its related parameters, such as number of individuals, number of replicates, mutation rate and demographic parameters; (1b) handling simulations: location on disk and filesystem layout; (2) preprocessing: initial data transformations, filtering values such as minimal number of sampled individuals or SNPs; (3a) architecture design: network name and related arguments (number of filters, layers,…); (3b) training: loss functions, optimization and training hyperparameters (number of epochs, learning rate, batch size, optimizer name,…); (3c) on-the-fly data transformations (subsampling, cropping,…). (**B**, **C**) Illustration of two standard use cases of dnadna. (**D**) Extract of a training configuration file in YAML format. (**E**) View of a plugin python file that will be passed to dnadnatrain, where users can implement novel networks based on PyTorch. **F**: Illustration of a regression task with a pre-trained network. Here we only need to use ‘dnadna predict’ since the network is already trained. Dotted lines denote true known histories, while boxplots indicate the population sizes predicted for 100 independent genomic regions at each time step. The estimates for a ‘complete’ genome are given by the averaged predicted values. The error corresponds to the relative squared errors averaged over the whole time period. (**G**) Illustration of a classification task, following Quickstart tutorial 2. The network was trained on a toy dataset (2000 independent population samples split into training and validation) and tested on 2000 additional samples to discriminate whether the population underwent a decline or an expansion of its size. The contingency table shows that for this classification task, the accuracy is 85.3% on a test set. See mlgenetics.gitlab.io/dnadna/tutorials.html for details on both experiments

Figure 1 provides an overview of dnadna steps. It is accessible without coding knowledge thanks to its YAML configuration files which provide the user with a variety of options at each step of the process. Because each use case has its own specificity, we developed a system which allows users to implement plugins (e.g. data transformations, simulators or networks) without modifying the core of the code.

dnadna is a Python (≥3.7) package with multiple dependencies, the main one being the open-source machine learning library PyTorch. It has a command line interface and an application programming interface (API) (mlgenetics.gitlab.io/dnadna/api.html). It is highly flexible thanks to a structured configuration file system based on YAML and JSON Schema. dnadna is dual-licensed under the GNU Lesser General Public License (LGPLv3+) and the compatible CeCILL-C Free Software License Agreement. Release 1.0 is available from PyPI at pypi.org/project/dnadna/ and Anaconda at anaconda.org/mlgenetics/dnadna. Docker images are available at hub.docker.com/u/mlgenetics.

## 3 Tutorial examples

We showcase various dnadna use cases via tutorials that will continue to expand. It is not required to have coding knowledge to perform similar tasks. A first tutorial walks the user through the complete process from configuring and generating simulated genetic data, to running data pre-processing and training a convolutional network to solve a regression task (here predicting the parameters of a two-step population size history). A second tutorial solves a classification task instead (Fig. 1G). A third tutorial helps users who already have simulated data, to familiarize themselves with dnadna and train a SPIDNA network on a provided dataset. Finally, we provide tutorials in the form of jupyter notebooks (mlgenetics.gitlab.io/dnadna/tutorials.html and Fig. 1F).

## Data Availability

The data underlying this article are available in its associated online software repository.
